# Data reduction for cough studies using distribution of audio frequency content

**DOI:** 10.1186/1745-9974-8-12

**Published:** 2012-12-12

**Authors:** Antony Barton, Patrick Gaydecki, Kimberley Holt, Jaclyn A Smith

**Affiliations:** 1 School of Electrical and Electronic Engineering, University of Manchester, Manchester, United Kingdom; 2 Respiratory Research Group, Manchester Academic Health Sciences Centre, University of Manchester, University Hospital of South Manchester, ERC Building, Second floor, Manchester, M23 9LT, United Kingdom

**Keywords:** Aacoustics, Cough sounds, Monitoring

## Abstract

**Background:**

Recent studies suggest that objectively quantifying coughing in audio recordings offers a novel means to understand coughing and assess treatments. Currently, manual cough counting is the most accurate method for quantifying coughing. However, the demand of manually counting cough records is substantial, demonstrating a need to reduce record lengths prior to counting whilst preserving the coughs within them. This study tested the performance of an algorithm developed for this purpose.

**Methods:**

20 subjects were recruited (5 healthy smokers and non-smokers, 5 chronic cough, 5 chronic obstructive pulmonary disease and 5 asthma), fitted with an ambulatory recording system and recorded for 24 hours. The recordings produced were divided into 15 min segments and counted. Periods of inactive audio in each segment were removed using the median frequency and power of the audio signal and the resulting files re-counted.

**Results:**

The median resultant segment length was 13.9 s (IQR 56.4 s) and median 24 hr recording length 62.4 min (IQR 100.4). A median of 0.0 coughs/h (IQR 0.0-0.2) were erroneously removed and the variability in the resultant cough counts was comparable to that between manual cough counts. The largest error was seen in asthmatic patients, but still only 1.0% coughs/h were missed.

**Conclusions:**

These data show that a system which measures signal activity using the median audio frequency can substantially reduce record lengths without significantly compromising the coughs contained within them.

## Introduction

Cough is the commonest symptom reported by patients to doctors and presents as part of the symptom complex of many respiratory diseases [1,2]. Until recent years the study of cough has been restricted by a lack of useful measurement tools, relying mainly upon subjective reporting of cough severity. The development of portable digital sound recording devices has allowed the number of cough sounds to be counted over extended time periods, providing an objective measure of cough rate and new insights into its determinants [3-6]. However, at present, sufficiently accurate algorithms are not in place to allow reliance upon fully automated detection systems. Patient recordings require laborious manual counting with confirmation of cough sounds by experienced observers. To enable studies of a meaningful size, either the present method of manual counting must be made more efficient, or for large studies an automatic system must be developed. The amount of data generated by larger cough studies requires either more trained human cough counters, or the computational power and algorithms to run an automatic system. Additionally, the constraints of battery life and integrated storage for a recording device can limit the scope of individual systems. A real-time algorithm embedded in a recording system which reduces the demand for data storage would reduce power consumption and increase the maximum possible record length achievable for such a system. Battery capacity remains a scarce resource for portable systems as longer battery lives in modern systems are enjoyed only as a result of such power demand reduction and not generally as a result of higher battery capacity. A system like this is essential for any medium to large study which seeks to make recordings of 24 hours or longer. Consequently, there is a need for a system which can minimise audio data prior to storage and cough counting in an effective manner without significantly affecting cough counts.

So far a number of projects have attempted to produce an automatic cough detection system [7], with limited success and no resultant commercially available system. Additionally, no research has been published which directly confronts the issue of data minimisation. The aim of this study is to provide the first of a series of modular elements of cough research software, which seeks to remove “inactive audio” from a patient recording. Inactive audio is defined as sections of a patient record which contain no sound that can be associated with cough and is not necessarily related to signal power. Simple approaches such as signal power thresholding are inappropriate for this application as cough sounds may not always be more powerful than inactive audio and quiet coughs may be of sufficiently low power to be rejected by a power threshold used alone. We have tried to overcome this by using the median frequency of the audio signal to provide a measure which is independent of signal power and can exploit the characteristic high frequency components of the cough sound.

We also aimed to address some of the difficulties in the assessment and reporting of the performance of automated cough detection systems. For example, the reporting of sensitivity, specificity and intra-class correlation coefficients may suggest systems perform accurately, when Bland-Altman plots of the same data suggest substantial errors in algorithm counts compared to the manual cough counts for individual subjects [8,9]. Two primary performance measures of the proposed system will be investigated in this study; the ability of the system to reduce audio record lengths and also the “destructiveness” of the system, i.e. the extent to which the system erroneously removes cough sounds and how this compares to the differences seen between experienced manual cough counters.

## Methods

### Subjects

We studied 20 subjects (10 male, 10 female), 5 each from groups of healthy volunteers (smokers and non-smokers), sufferers of chronic cough, chronic obstructive pulmonary disease (COPD) and asthma, see Table 1. Patients were recruited from the respiratory out-patient clinics at University Hospital South Manchester (UK) and healthy volunteers from hospital staff. Those taking Angiotensin Converting Enzyme inhibitors, opiates (or other anti-tussives), with significant co-morbidity or with a recent respiratory tract infection (<4 weeks) were excluded. Ethical approval was obtained from the Local Research Ethics Committee and all subjects provided written consent.

**Table 1:** Subject characteristics

	**Whole Group**	**Healthy/Healthy Smoker**	**Asthma**	**Chronic Cough**	**COPD**
**N (female)**	20 (10)	5 (3)	5 (3)	5 (3)	5(1)
**Age (SD)**	57.4 yrs (±10.9)	54.0 yrs (±10.5)	57.4 yrs (±4.4)	50.4 yrs (±14.4)	67.6 yrs (±4.0)
**Current Smokers**	3	3	0	0	0
**Smoking History pack years**median (range)	3.5 (0-40)	7.5 (0-29)	0 (0-5)	0 (0-2)	30 (28-40)
**FEV**_**1**_**% predicted (SD)**	86.1% (±27.3)	98.7% (±9.6)	102.2% (±11.8)	99.5% (±12.3)	44.1% (±14.0)
**FEV1/FVC % ratio (SD)**	66.3% (±15.6)	70.8% (±7.0)	73.6% (±4.5)	76.4% (±4.7)	44.4% (±15.5)

*FEV1* = forced expiratory volume in 1 second; *FVC* = forced vital capacity.

### The recording system and manual cough counting

The recording system used was the VitaloJAK^TM^ (Vitalograph Ltd, Buckingham, UK), a two channel 24 h recording device with two sensors; one free air condenser microphone and one chest wall air-coupled condenser microphone. Recordings are made at a sample rate of 8 kHz and bit rate of 16 bits per sample and stored on a compact flash memory card.

Twenty-four hour recordings were made for all 20 patients, each of which was broken up into 15 minute segments for analysis. Each 15 minute segment was subsequently counted by trained manual cough counters (n = 3) and labelled with a number of coughs (Count A). We have previously demonstrated an excellent level of agreement between trained manual cough counters in a variety of diseases [6,10,11]. Additionally, 15 minute segments with zero cough counts were identified and excluded from testing the destructiveness of the algorithm as they would provide no useful information but still included in the data reduction analysis.

### The algorithm

**Figure 1:**
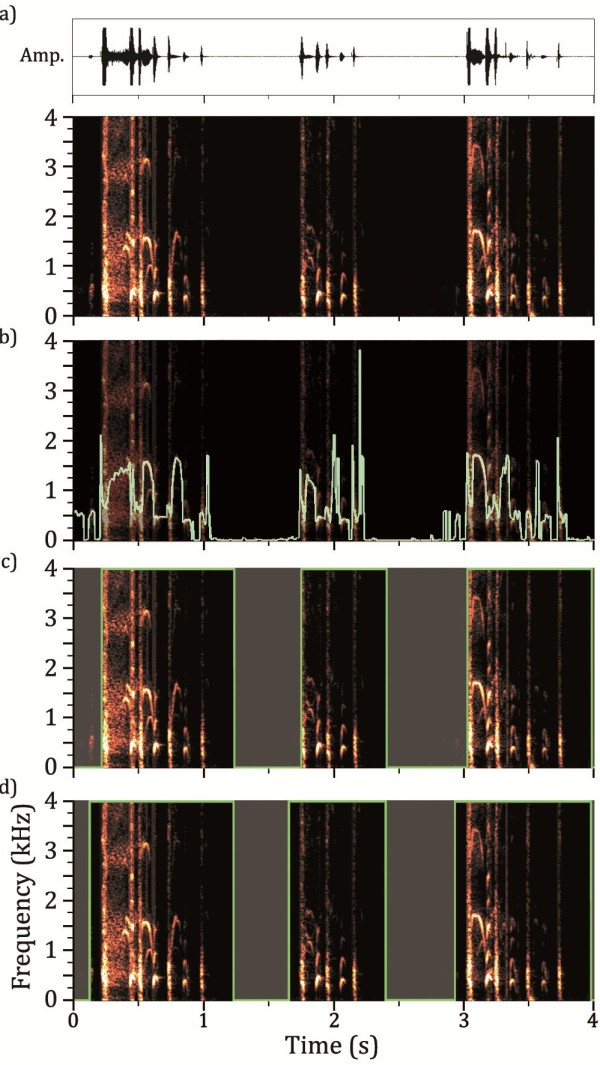
**Spectrograms of a 4 second segment of audio, containing a series of coughs, showing stages of algorithm; a) 512 sample Fast Fourier Transform of audio data is taken every 16 samples and positive half of spectrum is plotted vertically using intensity of colour as contribution of frequency to signal.** This spectrogram is shown underneath the audio waveform. **b**) Median frequency is calculated for each point in spectrogram and shown here superimposed. **c**) Threshold value for median frequency used to generate a ‘mask’ indicating which audio is to be removed, indicated graphically here as the faded sections. **d**) Timings for cuts of audio data adjusted to ensure ‘attack’ of sounds is captured.

Each of the 15 minute segments were processed separately by the algorithm implemented in MATLAB (Mathworks Inc., Natick, MA, USA). Each recording was processed as a set of 512 sample Fast Fourier Transforms (FFTs) taken in 16 sample steps. The Fourier Transform of a signal breaks the signal down into a sequence of numbers representing the activity of evenly spaced frequency bands. A Fast Fourier Transform (FFT) is an optimised form of the Fourier Transform which executes in a much reduced period of time. The transform loses all information about the timing of the signal (e.g. when particular frequency components start or end), but the resultant transforms calculated from a series of overlapping and short segments of audio when laid side by side can be assembled into a ‘spectrogram’ (Figure 1a). This spectrogram shows the variation of frequency content of the recorded sound against time, usually using colour intensity to indicate the contribution of a particular frequency at a given time. The sum and median of the numbers from each transform were then calculated to produce a ‘signal activity profile’ for the record which can be superimposed on the spectrogram as a visual aid (median shown in Figure 1b). The sum was used as an additional measure to avoid the system treating quiet audio as active due to the median rising on very low power signals, which will mostly contain Gaussian (white) noise, and have a median frequency of half the maximum frequency (i.e. a quarter of the sample rate of the recording). A threshold is then applied for both the median frequency and integral for each point to generate a ‘mask’ (i.e. a map of which audio is to be kept and which is to be removed) to apply to the audio recording (Figure 1c). The sections of the mask which keep audio are then expanded forwards and backwards in time to ensure the attack and decay of individual sounds are retained in full and any chopping effects due to very small sections of rejected audio are eliminated (Figure 1d). For the purposes of this investigation ‘positive’ and ‘negative’ records were generated, representing the audio kept and the audio removed respectively. Operation of the algorithm is fully automated; the software is given the location of the source audio files and produces the divided positive and negative records with no user intervention.

### Algorithm validation

#### Assessment of file reduction

To measure the extent of audio reduction, the algorithm was used to process all of the 24 h recordings from the 20 patients and the positive records were produced to determine the length of the remaining audio. All the data were used as this provides a comprehensive measure of the reduction in storage requirements and duration of file for manual counting that the algorithm would generate. The data were then analysed both as individual segments and as full 24 h recordings to identify the level of reduction achieved.

#### Assessment of destructiveness

The value of the algorithm depends upon retention of an acceptable proportion of coughs from patient records. This can only be established by comparison with manual cough counts, despite such counts having an intrinsic variability. In our experience, the average agreement between manual cough counts is usually <1 cough/h and varies by approximately ±2 coughs/h (95% limits of agreement) [6,10,11]; thus an alternative approach to assessing algorithm performance is to compare the variability introduced by the algorithm to the intrinsic variability seen between manual counts. We find manual counts vary due to inconsistency from human error and from variable inter-observer interpretation of ‘borderline’ sounds (e.g. very quiet cough sounds and sounds difficult to classify as cough or throat clearing), where the algorithm will be more consistent, i.e. the same sound will always produce the same result.

To determine the magnitude of destructiveness, the algorithm was tested using 200 segments from 20 patients (34 healthy, 60 chronic cough, 54 COPD, 52 Asthma). Segments with no coughs counted by the initial manual counts (Count A) were eliminated from this test before selection of segments as they would provide no useful measure. Clearly for the healthy patients this results in a minimal number of useful segments such that there were fewer than 10, in this case the deficiencies in each individual were supplemented by random extra segments from other patient groups.

Once the segments were selected and processed, the positive and negative records were then returned to a single manual counter for counting (Count B and C respectively) in the same manner as unprocessed segments; the counter was blinded to the original counts (A). To cope with the potential for parts of a single cough to occur on both records (positive and negative), the manual counter was instructed to count only the start of a cough, i.e. its explosive phase. Ideally, the negative records should contain no coughs (C = 0) and the positive records should contain the same number of coughs as initially counted (B = A). Any coughs counted in negative records are referred to as ‘missed’ coughs. The total of both counts (B + C = D) was also compared to the original (A) as an indication of variability introduced by manual counting.

### Statistical analysis

The different cough counts used in the statistical analysis are referred to by letter as follows:

•Count A: Original counts of 15 minute segments

•Count B: Counts from ‘positive’ reduced audio segments.

•Count C: Counts from ‘negative’ reduced audio segments, also referred to as ‘missed coughs’.

•Count D: Sum of counts from respective positive (B) and negative (C) segments for each 15 minute segment

#### Assessment of file reduction

For record lengths, we calculated the mean (standard deviation) as a percentage (%), duration (minutes) and skewness of the resultant length of both 15 minute segments and whole 24 h records. The minimum recording time required to capture a 24 h period for a given proportion of random recordings was estimated as a measure of the performance of the reduction with respect to change in demand on the recording hardware.

The t-distribution to estimate the statistics of patient recordings based on the data collected along with basic statistical measures such as mean, standard deviation and skewness.

#### Assessment of destructiveness

For destructiveness we calculated the mean missed cough rate per hour (coughs/hr) and the proportion of coughs missed (%) overall and per patient group, the average difference and the average rate of deviation (i.e. the absolute magnitude of the difference) from manual counts (coughs/hr) per patient group and overall. The deviation of cough counts from the original is the absolute value of the sum of positive and negative files (D) minus the original count (A) which can be represented as Abs(D-A).

The difference and the deviation in manual cough counts from the original files and both the algorithm processed files (B + A) was also calculated to provide a measure of variability between manual coughs.

## Results

### Assessment of file reduction

**Figure 2:**
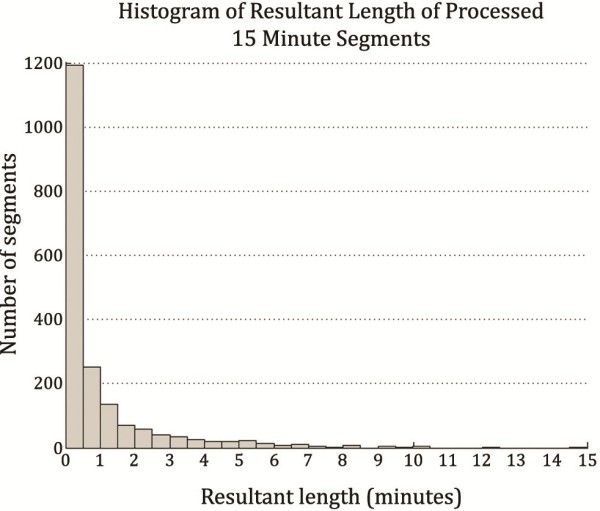
**Histogram of the file lengths of 1920 segments processed by the algorithm.** The overwhelming majority of segments have been reduced to files of length below 30 seconds.

The mean resultant 15 minute segment length was 6.04% of the original size (54.4 seconds ±96.6) (median 0.23 minutes, IQR 0.94 minutes). As implied by the relative magnitude of the mean and standard deviation there is a very strong positive skew (3.25) in the resultant length of individual segments (Figure 2). The mean resultant length for whole 24 h patient records was 6.25% (87.0 minutes ±68.3) (median 62.4 minutes, IQR 100.4 minutes). Again, the data show a positive skew (0.95) but much smaller in magnitude than for the individual segments.

A t-distribution with 19 degrees of freedom was used to estimate the expected minimum reduction across a given proportion of patients using the sample standard deviation and mean from the resultant lengths of the 20 full patient records. The estimated model showed that 99.9% of patient records would see a reduction down to at worst 23.1% (5.54 hours) and 95% of patient records would achieve better than down to 14.2% (3.41 hours) of original size.

### Assessment of destructiveness

The total cough counts for the files analysed by diagnosis and gender are shown in Table 2 with the resulting median cough rates per subject in Table 3. For the majority of subjects and diagnoses very few coughs were missing from the positive files and therefore the cough rates for the positive files are very similar to those for the full files (rate B versus rate A), with no statistically significant differences overall (p = 0.44). Most of the coughs missed were removed from the recording of a single female asthma patient, causing the relatively unfavourable results for both the female and asthmatic categories.

**Table 2:** Cough counting results

**Patient group**	**Number of patients**	**Cough count for full files (A)**	**Positive file count (B)**	**Negative file count/Missed coughs (C)**
**Healthy/healthy smoker**	5	219	220	0
**Chronic cough**	5	1228	1244	1
**COPD**	5	148	140	1
**Asthma**	5	337	293	29
**Male**	10	584	572	2
**Female**	10	1348	1325	29
**Overall**	**20**	**1932**	**1897**	**31**

**Table 3:** Cough counts expressed as coughs per hour per patient; data are median (inter-quartile range)

	**Overall**	**Healthy volunteers**	**Chronic cough**	**COPD**	**Asthma**
	**(n = 20)**	**(n = 5)**	**(n = 5)**	**(n = 5)**	**(n = 5)**
**Full File** Cough Rate (A)	26.8c/h (13.1-51.6)	26.0c/h (16.8-53.6)	77.3c/h (43.7-117.5)	10.0c/h (7.3-15.4)	27.6c/h (13.4-36.2)
**Positive File** Cough Rate (B)	25.2c/h (12.1-51.8)	26.0c/h (17-53.6)	77.3c/h (44.5-119.3)	8.8c/h (7.5-14.6)	24.4c/h (13.2-30.0)
**Negative File** Cough Rate (C)	0.0c/h (0.0-0.2)	0.0c/h (0.0-0.0)	0.0c/h (0.0-0.2)	0.0c/h (0.0-0.2)	0.4c/h (0.0-4.8)
**% missed cough/h**	0.0% (0.0-0.1)	0.0% (0.0-0.0)	0.0% (0.0-0.1)	0.0% (0.0-0.7)	1.0% (0.0-4.3)

Note that only segments containing cough were selected for this analysis therefore cough rates are not typical of those seen for full 24 hr recordings in these disease groups

Table 4 shows the median differences (IQR) and absolute deviations of the algorithm cough rate from the full file cough rates, showing both are relatively low except for in the asthma patients. The same calculations have also been performed for the cough rates counted from the full files compared to the positive plus negative files, providing the differences and deviations between manual cough counters for comparison. These were not significantly different from those for the algorithm (p = 0.06 and p = 0.28 respectively).

**Table 4:** Comparison of cough rates counted from positive algorithm files with original full files and comparison of cough rates counted from positive plus negative files with original full files; all data are median (inter-quartile range)

	**Overall**	**Healthy volunteers**	**Chronic cough**	**COPD**	**Asthma**
	**(n = 20)**	**(n = 5)**	**(n = 5)**	**(n = 5)**	**(n = 5)**
**Algorithm Difference** (B-A)	0.0c/h (-1.2 to 0.4)	0.0c/h (0.0 to 0.2)	0.0c/h (-0.2 to 2.8)	0.0c/h (-2.5 to 1.4)	-3.2c/h (-6.2 to -0.2)
**Manual Counters Difference** (D-A)	0.0c/h (-0.4 to 0.4)	0.0c/h (0.0 to 0.2)	0.3c/h (-0.2 to 2.8)	0.0c/h (-2.5 to 1.6)	-0.4c/h (-3.2 to 0.4)
**Algorithm Absolute deviation** (B-A)	0.6c/h (0.0 to 2.9)	0.0c/h (0.0 to 0.2)	0.4c/h (0.0 to 2.8)	1.3c/h (0.4 to 2.8)	3.2c/h (0.6 to 6.2)
**Manual Counters Absolute deviation** Abs(D-A)	0.4c/h (0.0 to 2.0)	0.0c/h (0.0 to 0.2)	0.36c/h (0.2 to 2.8)	1.3c/h (0.6 to 2.8)	0.4c/h (0.4 to 3.2)

## Discussion

This study demonstrates that median frequency analysis can reduce 24 h audio records down to a median of just over 1 h in length, and the overall variability in the resultant cough counts is comparable to the current gold standard, which is seen between trained manual cough counters. It has also been estimated that for 99.9% of subjects the system can be expected to reduce data down to less than a quarter of its original size. The system can also be executed faster than real-time allowing it to be directly integrated to a recording system. With these results it is clear that this system can be of great use for the purposes of extending the recording time capabilities of a cough recording system and minimising data for storage prior to either manual or as an intermediate part of an automatic cough counting system.

Interestingly, median frequency analysis is not uncommonly used in the spectral analysis of other biomedical signals, such as EMG [12] and EEG [13], and has also been applied to the assessment of lung sounds. In asthma patients, changes in the median frequency of breath sounds reportedly correlates with changes in airway flows during bronchoconstriction [14,15]. As coughing is typically associated with sudden increases in flow, this may in part explain the utility of this algorithm for identifying potential cough sounds.

The destructiveness of the algorithm was well within the previously reported tolerance of ±2coughs/hr for mean difference and also, more importantly, comparable to the difference between manual cough counters in this study (i.e. the original manual counts (A) and counts of positive and negative files combined (D)). This implies that the discrepancy between counts of processed and unprocessed data will mostly be explained by the disagreement in manual counts, rather than due to coughs erroneously removed. The reduced length of audio records may also help manual counters by reducing the challenge of maintaining full attention for long periods of time.

The majority (81%) of coughs missed by the algorithm were missed from one patient. Upon inspection of the raw data and measurement of the coughs missed, these coughs sounded muffled, as if either the mouth was closed or obstructed by a hand or clothing. They were low in volume causing the algorithm to reject them both on the grounds of being below the very low signal power threshold and below the median frequency required to prevent data removal. Due to the rarity of such incidents this would seem unlikely to be a significant problem and is not indicative of a particular problem with algorithm performance in asthma.

Recent publications suggest that automated/semi-automated cough detection systems may introduce significant errors into the resulting cough counts. As these errors randomly include both over-counting and under-counting, the average difference between algorithm counts and manual counts is often close to zero, but contain errors individual cough counts of ±40coughs/hr [8] and ±2coughs/minute [9]. We suggest the method described here, comparing the variability introduced by any process used for cough detection with that intrinsic between trained cough counters (i.e. the gold standard), gives a more useful assessment of algorithm performance than those previously described [16]. In addition calculation of the absolute deviation of algorithm counts from manual counts gives a more transparent quantification of errors.

Calculating resultant length of 24 h records using 20 patients, only allows for a conservative estimate and in future more patients should be studied to gain a clearer idea of how they vary. However, 20 patients is a relatively large number of patients for a cough related system validation [8,9,17] and highly demanding on manual counting resources to support findings. Moreover, a broader range of conditions was included than in other studies, suggesting this algorithm is robust to any differences in cough acoustics between different diseases. The disadvantage of this approach is that only small numbers of subjects were included for each diagnosis, allowing individual results to have undue influence, such as in our asthma data.

## Conclusion

This study has demonstrated an algorithm based upon a median frequency threshold is capable of substantially shortening 24 h cough sound recordings with minimal loss of cough data. In recent years we have shown that the objective measurement of cough sounds is not only important in understanding the effects of anti-tussive agents [18,19] but also provides novel insights into the mechanisms determining chronic coughing [5,6]. This is an important development, not only facilitating the manual counting of cough sounds, currently the most accurate method for quantifying cough frequency, but also providing an essential step towards the development of more accurate automated algorithms.

## Abbreviations

FFT: Fast Fourier Transform; Abs(x): The absolute value of x (i.e. always positive).

## Competing interests

JAS is an inventor on a patent describing methods of cough detection and has a collaboration with Vitalograph Ltd to develop a commercial cough monitoring system.

## Authors’ contributions

AB & JAS participated in preparation and finalisation of manuscript, KH carried out data collection and PG participated in finalisation of manuscript. All authors have read and approved the final manuscript.
